# Degradable Polyurethane Foams Based on Amino Acid Phosphoramides (APtA)

**DOI:** 10.3390/polym18121534

**Published:** 2026-06-20

**Authors:** Nico Vennemann, Anton Bauer, Oliver Brüggemann

**Affiliations:** Institute of Polymer Chemistry, Johannes Kepler University, 4040 Linz, Austria

**Keywords:** polyurethanes, degradable polymers, phosphorous compounds, PU foams

## Abstract

Soft polyurethane foams are commonly found in furniture, mattresses, shoes and soundproofing applications. These crosslinked foams are hard to recycle. This paper describes our approach of introducing chemical breakage points based on amino acid phosphoramidates (APtA) in the PUs’ backbones. The APtA monomers are combined with PEG, PPG and pTHF-chains to achieve different monomer structures. We demonstrate the hydrolysis of these APtA monomers at neutral pH 7 and the mass loss of the foams. It is shown that after 70 days, more than 50% of the p-THF-APtA monomer and 35% of the PPG-APtA monomer have degraded. However, a contrary trend was observed for the foams, with only 2.5% mass loss for the p-THF-APtA foam, but 26% mass loss for the PPG-APtA foam. The foams were also characterized using compression measurements, revealing a stiffer appearance of the p-THF-APtA foam compared to the foams based on PEG and PPG. SEM images were taken before and after the degradation of the foams to show the difference in morphology.

## 1. Introduction

Soft polyurethane foams (PUR foams) are commonly found in furniture, car seats, shoes, padding and soundproofing applications [1]. Although they are quite common in everyday applications, most soft foams are not biodegradable and can only be partly recycled, e.g., as filler materials [2,3,4]. Chemical recycling, such as acidolysis, pyrolysis or aminolysis are possible, but are not used on industrial scale [4,5,6].

There are already different publications on the topic of degradable polyurethane foams. Savelyev’s work shows the application of disaccharides as degradable linkers [7]. Serrano achieves degradable foams by using a mixture of castor oil and polyesters obtained from wheat straw (mostly cellulose and hemicellulose) [8]. Huan et al. synthesized biodegradable polyurethane fibers based on 2-aminoethanol and α,ω-carboxylic acids that slowly hydrolyze [9]. Other works also show the possibility of degradable polyurethanes by either employing hydrolysis, photodegradation, heat or microorganisms in order to degrade the (poly)ester and urethane bonds [10,11,12,13,14].

However, all these approaches work by replacing the ethylene oxide (EO) and propylene oxide (PO)-based polyols, found in most soft PUR foams, with different types of polyols, mostly polyesters. This leads to the PUR foams possessing different mechanical properties and different characteristics. Previous work has shown that it is possible to insert chemical breakage points into polyurethanes. Amino acid phosphorodiamidates (APdA) have been successfully integrated into linear PU chains and show the degradation of these TPUs under mild conditions [15].

**Figure 1:**
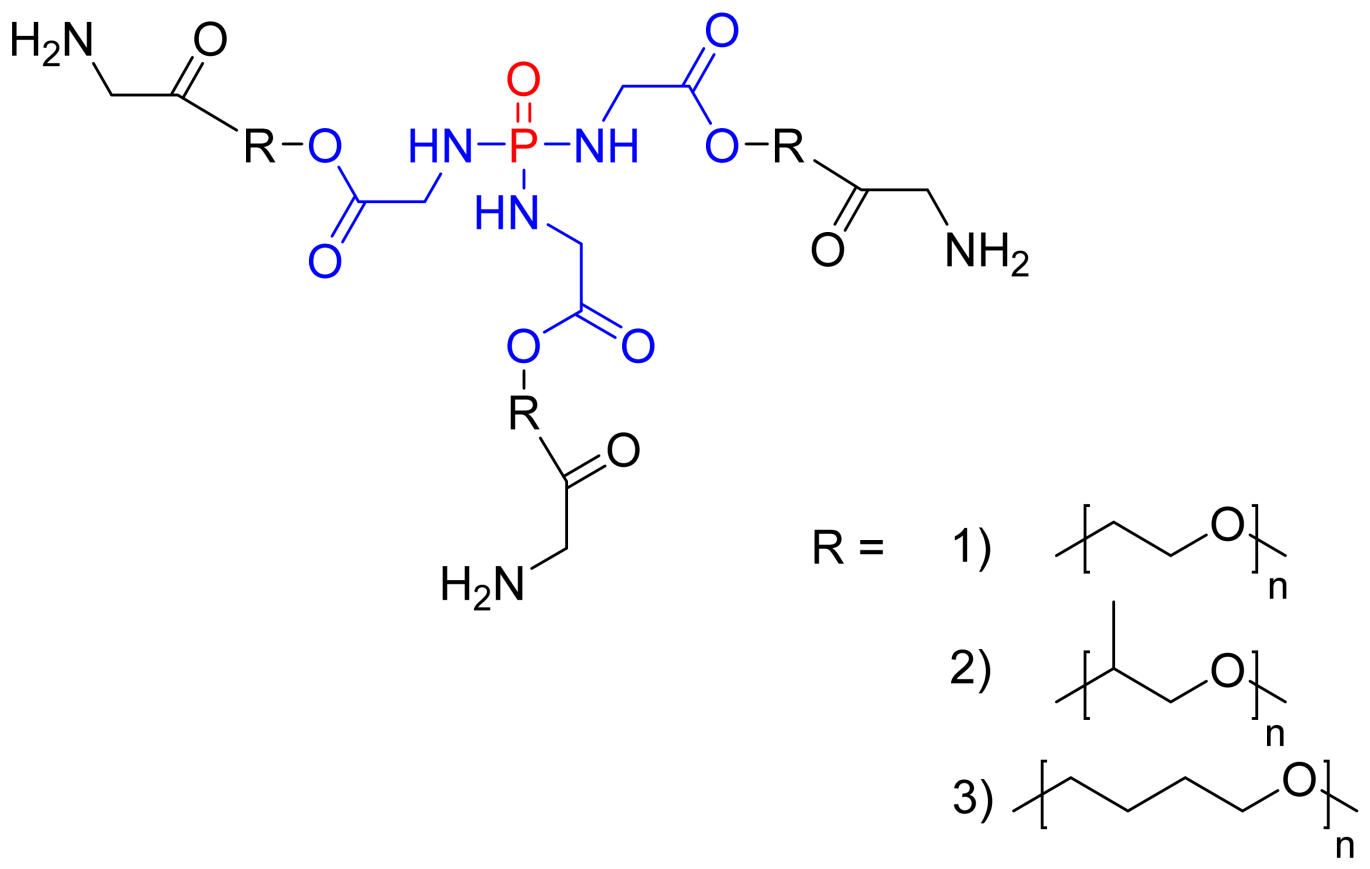
APtA monomers with different polyol arms (1) PEG (2) PPG (3) pTHF, with the amino acid based chemical breakage point highlighted in blue and its core colored in red.

Similar to the difunctional APdA monomers, trifunctional amino acid phosphoramides (APtA) can be used as trifunctional crosslinkers. The amino acid plays a crucial role in the degradability of the APdA and APtA units. While other phosporamides degrade slowly or only under acidic conditions, APtA degrade fast and at a neutral pH. The used amino acid also has a considerable effect on the degradation behavior. Glycine-based monomers degrade faster than alanine or valine-based APtA. This was proven previously by our group [16,17]. The foams described in this paper are based on polyether-based amino acid phosphoramides, which are trifunctional monomers with high molecular mass. 4,4-Methylene diphenyl diisocyanate (MDI) is used as isocyanate and water as a foaming agent. Three different polyols (PEG, PPG, pTHF), with a molecular weight of 1000 g mol^−1^ each, have been used to synthesize three different degradable APtA-based monomers. The structure of the APtA-based monomers is shown in Figure 1. The polyols have been end-capped with glycine to achieve a facile synthesis and fast degradation of the APtA-based monomers.

**Figure 2:**
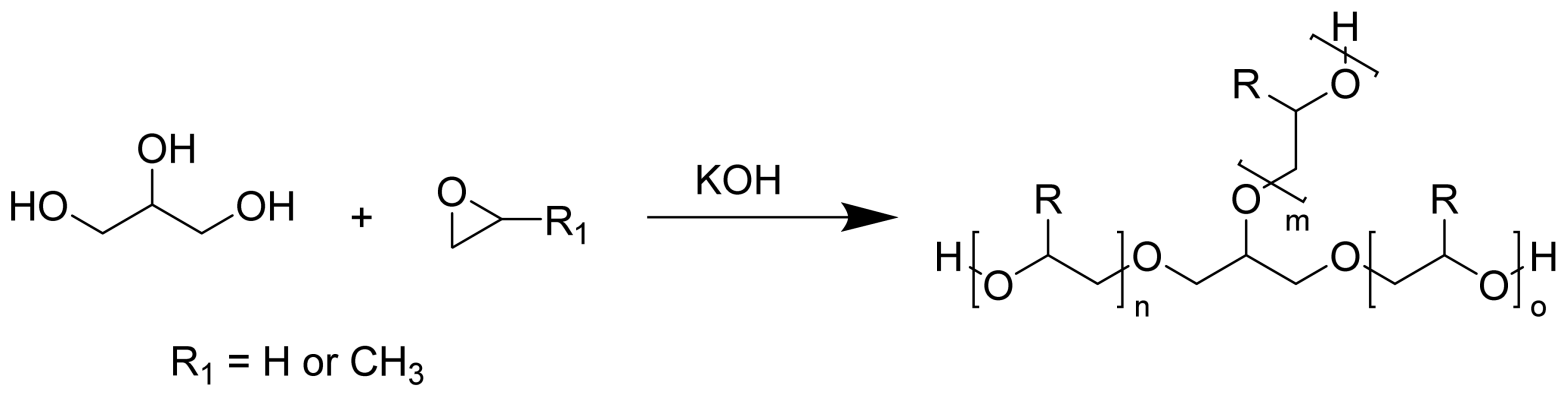
Synthesis of polyether-based polyols for soft PU foams, using glycerol as starting molecule linked to ethylene oxide (R=H) and/or propylene oxide (R=CH_3_).

Most soft PUR foams are based on trifunctional (or higher) monomers, such as glycerol. The hydroxy functionalities of the starter monomer are then reacted either with EO or PO, or a mixture of both of them, to yield a higher molecular weight trifunctional monomer. The reaction is shown in Figure 2. The reason for using EO or PO is that they offer good material properties and are readily available. EO is more polar and offers higher reactivity (because of primary hydroxyl groups), while PO offers better flexibility [18,19]. The molecular weight of the monomers is mostly between 2000 and 5000 g mol^−1^, and they contain three hydroxyl groups. The most common isocyanates are MDI or toluene diisocyanate (TDI), as both offer high reactivity and foam stability.

**Figure 3:**
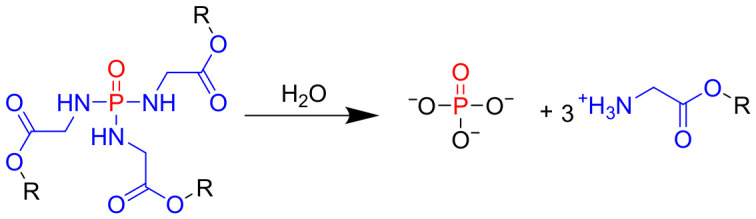
Hydrolytic degradation of amino acid phosphoramide (APtA) used as degradable monomer for the synthesis of soft PUR foams, with the amino acid based chemical breakage point highlighted in blue and its core colored in red. R equals the polyol backbone and/or foam structure.

In our approach, a chemical breakage point is inserted into these polyols by replacing the common starter molecule of glycerol with a phosphoramide, keeping the EO and PO-based structures, using polyethylene glycol and polypropylene glycol, respectively (Figure 1, (1) + (2)), and thus combining the great material properties of polyether polyols, while gaining a degradable monomer. The P-N bond can be hydrolytically cleaved at a neutral pH, but degrades faster under acidic conditions [15,17]. Figure 3 shows the degradation mechanism of the APtA monomers upon hydrolysis. The degradation seems to follow a one-step degradation, as there were no intermediate signals detected using ^31^P-NMR spectroscopy. This is in contrast to the APdA from previous works, where an intermediate degradation product could be observed [15,17]. Upon cleavage of the P-N bond, only linear chains, comprising the glycine–polyol ester and MDI units, as well as phosphates, are left. As the crosslinking points degrade, the foam is expected to slowly lose its mechanical properties and collapse.

## 2. Materials and Methods

### 2.1. Chemicals

All chemicals were bought at several suppliers and were used as received. 4,4 Methylene diphenyl diisocyanate was purchased from THERMO Sci (St. Peter Straße 25, 4020 Linz, Austria). Dibutyltin dilaurate and 4 M dioxane HCl were bought from TCI (Mergenthaleralle 79-81, 65760 Eschborn, Germany). Triethylamine, glycerol propoxylate (M_n_ = 266) and glycerol propoxylate block ethoxylate (M_n_ = 5300), HEPES, p-toluene sulfonic acid monohydrate, polytetrahydrofuran (M_w_ = 1000) and phosphoryl bromide were purchased from Sigma Aldrich (Marchettigasse 7, 1060 Vienna, Austria). MgSO_4_ was bought from VWR chemicals (Gen. Sowińskiego 11, 44-121 Gliwice, Poland). Boc-Glycine and polyethylene glycol (M_w_ = 1000) were purchased from BLD pharmatech (Senefelder-Ring 27, 21465 Reinbek, Germany). Polypropylene glycol (M_w_ = 1000) was received from Carl Roth. All solvents were obtained from FischerSci (Dresdner Straße 89, 1200 Vienna, Austria).

All NMR spectra were recorded with a Bruker Avance III 300 (Bruker Austria GmbH, Lemböckgasse 47b, 1230 Vienna, Austria) and referenced to the signal of internal CDCl_3_ or DMSO-d_6_ purchased from Deutero (Am Ring 29, 56288 Kastellaun, Germany). ^1^H NMR spectral data are given in δ/ppm relative to residual solvent peaks.

### 2.2. Synthesis

#### 2.2.1. Glycine-Polyethylene Glycol (Gly-PEG)

**Scheme 1:**
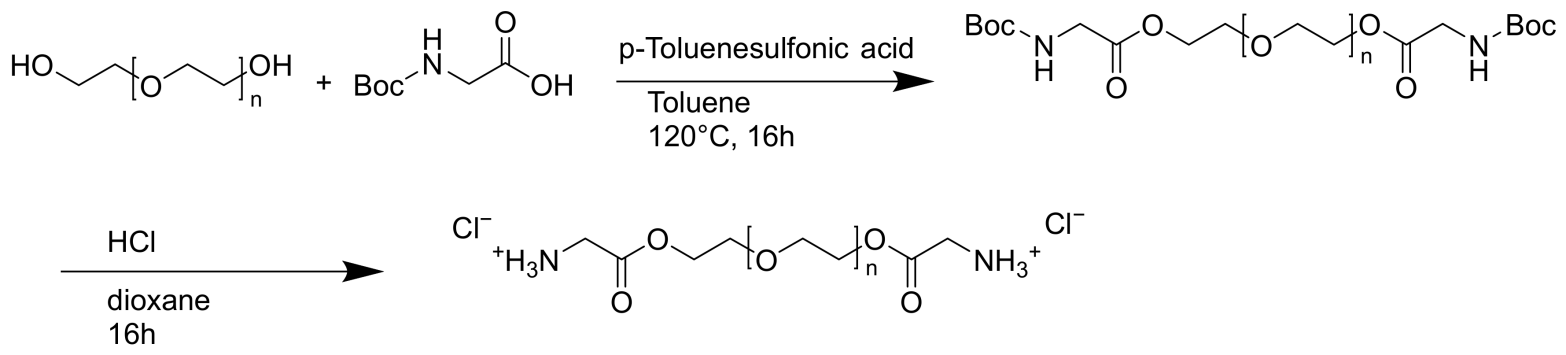
Synthesis of Gly-PEG via Fischer esterification using PEG1000 and Boc-Glycine, followed by deprotection using HCl.

Boc-Gly-OH (18.4 g, 0.105 mol, 3.5 eq.) and PEG1000 (30 g, 0.030 mol, 1 eq.) were dissolved in 200 mL toluene and p-toluene sulfonic acid monohydrate (0.285 g, 1.5 mmol, 0.05 eq.) was added (Scheme 1). The reaction was refluxed under inert Dean–Stark conditions at 120 °C for 16 h. After completion, the solvent was removed and the product was taken up in cold diethyl ether and filtered. The product was washed three more times with cold diethyl ether, yielding the product Boc-Gly-Peg.

For the deprotection, Boc-Gly-Peg was dissolved in 40 mL 1,4-dioxane and 40 mL HCl (4 M in dioxane) was added. The reaction was stirred at RT overnight and the solvent was removed, yielding Gly-PEG as a dark orange oil (yield 67%) (Table 1).

^1^H-NMR (300 MHz, CDCl3, δ/ppm):

**Table 1:** ^1^H-NMR shifts for Gly-PEG.

Shift	Integration	Multiplicity	Designation
3.43–3.49	160H	m	O-C*H*2C*H*2-O
3.96–3.98	4H	m	N-C*H*_2_
4.37–4.39	4H	m	OCO-C*H*_2_
8.34	6H	s	N*H*_3_^+^

#### 2.2.2. Glycine-Polypropylene Glycol (Gly-PPG)

**Scheme 2:**
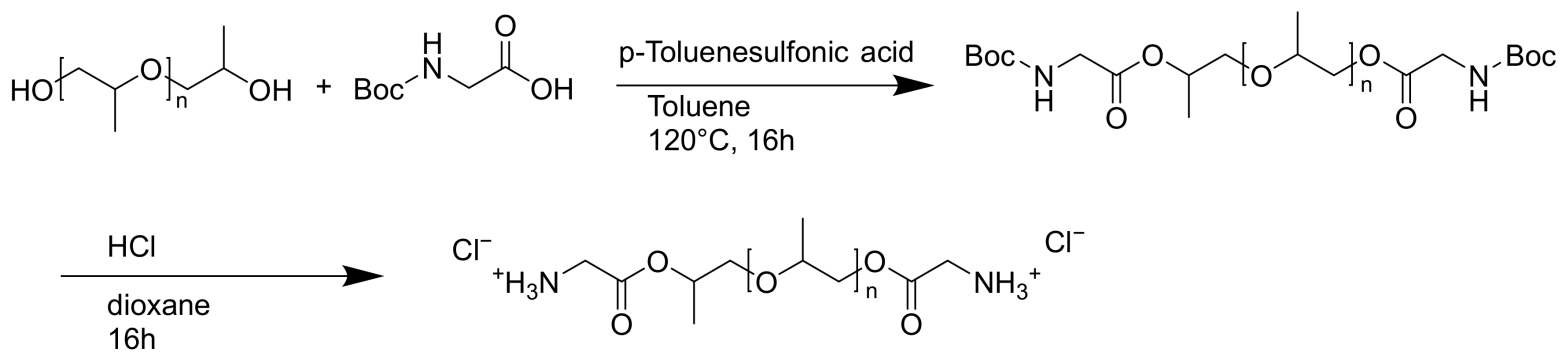
Synthesis of Gly-PPG via Fischer esterification using PPG1000 and Boc-Glycine, followed by deprotection using HCl.

Boc-Gly-OH (18.4 g, 0.105 mol, 3.5 eq.) and PPG1000 (30 g, 0.030 mol, 1 eq.) were dissolved in 200 mL toluene and p-toluene sulfonic acid monohydrate (0.285 g, 1.5 mmol, 0.05 eq.) was added (Scheme 2). The reaction was refluxed under inert Dean–Stark conditions at 120 °C for 16 h. After completion, the solvent was removed and the product was taken up in DCM and washed with NaHCO_3_ trice and once with brine. The product was dried over MgSO_4_ to yield Boc-Gly-PPG as orange liquid.

For the deprotection, Boc-Gly-PPG was dissolved in 40 mL 1,4-dioxane and 40 mL HCl (4 M in dioxane) was added. The reaction was stirred at RT overnight and the solvent was removed, yielding Gly-PPG as a viscous orange liquid (yield 76%) (Table 2).

^1^H-NMR (300 MHz, CDCl_3_, δ/ppm):

**Table 2:** ^1^H-NMR shifts for Gly-PPG.

Shift	Integration	Multiplicity	Designation
1.08–1.16	51H	m	-C*H*_3_
3.25–3.70	55H	m	C*H*, N-C*H*_2_-COO, C-C*H*_2_-O
8.55	6H	s	N*H*_3_^+^

#### 2.2.3. Glycine-Poly-Tetrahydrofuran (Gly-pTHF)

**Scheme 3:**
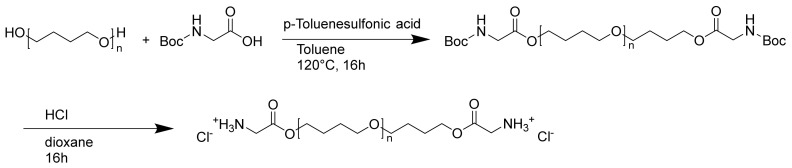
Synthesis of Gly-pTHF via Fischer esterification using pTHF1000 and Boc-Glycine, followed by deprotection using HCl.

Boc-Gly-OH (18.4 g, 0.105 mol, 3.5 eq.) and pTHF1000 (30 g, 0.030 mol, 1 eq.) were dissolved in 200 mL toluene and p-toluene sulfonic acid monohydrate (0.285 g, 1.5 mmol, 0.05 eq.) was added (Scheme 3). The reaction was refluxed under inert Dean–Stark conditions at 120 °C for 16 h. After completion, the solvent was removed and the product was taken up in ethyl acetate (200 mL) and washed with Na_2_CO_3_ twice (2 × 100 mL) and once with brine (100 mL). The ethyl acetate was dried over MgSO_4_ and the solvent removed to yield Gly-pTHF as a white wax-like solid.

For the deprotection, Boc-Gly-pTHF was dissolved in 40 mL 1,4-dioxane and 44 mL HCl (4 M in dioxane) was added. The reaction was stirred at RT overnight and the solvent was removed, yielding Gly-pTHF as a colorless oil (Yield 92%) (Table 3).

^1^H-NMR (300 MHz, CDCL_3_, δ/ppm):

**Table 3:** ^1^H-NMR shifts for Gly-pTHF.

Shift	Integration	Multiplicity	Designation
1.56	28H	m	C-C*H*_2_-C
3.34	26H	m	C-C*H*_2_-O
3.89	4H	s	OOC-C*H*_2_-N
4.13	4H	t (J = 6.12 Hz)	C-C*H*_2_-COC
8/8.51	6H	s	N*H*_3_^+^

#### 2.2.4. APtA Synthesis

**Scheme 4:**
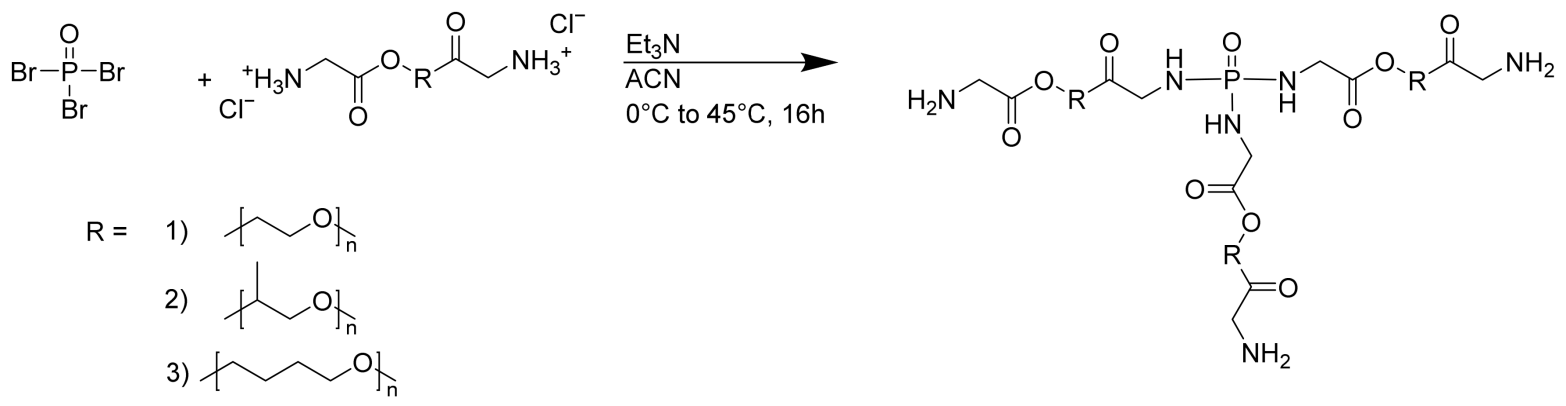
Synthesis of APtA with (1) Gly-PEG, (2) Gly-PPG and (3) Gly-pTHF.

For the synthesis of the APtA-Polyols, the previously synthesized Gly-PEG, Gly-PPG or Gly-pTHF (20.0 g, 0.017 mol, 3.05 eq.) were added to anhydrous acetonitrile and cooled to 0 °C under inert conditions (Scheme 4). Triethylamine (7.05 mL, 0.050 mol, 9.10 eq.) was added. Phosphoryl bromide (1.43 g, 5 mmol, 1 eq.) was dissolved in anhydrous acetonitrile and slowly added to the reaction. After the addition of the phosphoryl bromide, the reaction was heated to 45 °C for 16 h. After completion, the solution was filtered and the solvent was removed. The precipitate was taken up in ethyl acetate (200 mL) and washed once with an Al_2_Cl_3_ solution (25 mL) and once with brine (25 mL). The organic phase was dried over MgSO_4_ and the solvent was removed, yielding the corresponding Gly-Polyol-APtA.

PEG-APtA (yield: 86%) (Table 4):

^1^H-NMR (300 MHz, CDCl_3_, δ/ppm):

**Table 4:** ^1^H-NMR shifts for PEG-APtA.

Shift	Integration	Multiplicity	Designation
3.32–3.98	640H	m	O-C*H*2C*H*2-O, N-CH_2_
4.19–4.22	12H	m	OCO-C*H*_2_

^31^P-NMR (121 MHz, CDCl_3_, δ/ppm): 15.39.

PPG-APtA (yield: 82%) (Table 5):

^1^H-NMR (300 MHz, CDCl_3_, δ/ppm):

**Table 5:** ^1^H-NMR shifts for PPG-APtA.

Shift	Integration	Multiplicity	Designation
1.08–1.16	154H	m	-C*H*_3_
3.25–3.70	167H	m	-C*H*-, N-C*H*_2_-COO, C-C*H*_2_-O

^31^P-NMR (121 MHz, CDCl_3_, δ/ppm): 15.44.

pTHF-APtA (yield: 76%) (Table 6):

^1^H-NMR (300 MHz, CDCl_3_, δ/ppm):

**Table 6:** ^1^H-NMR shifts for pTHF-APtA.

Shift	Integration	Multiplicity	Designation
1.29–1.74	348H	m	OC*H*_2_C*H*_2_O
3.00–3.63	343H	m	N-C*H*_2_
3.86	3H	s	N_H_
4.01	12H	t (J = 6.2 Hz)	OCO-C*H*_2_

^31^P-NMR (121 MHz, CDCl_3_, δ/ppm): 15.52.

The monomers were stored in vials under argon.

### 2.3. Synthesis of Foams

Foam synthesis was adapted from the study by Pinto [20]. A reference foam was synthesized in addition to foams containing the degradable monomers Gly-PEG-APtA, Gly-PPG-APtA and Gly-pTHF-APtA. For this, 2.0 g of the monomer was mixed with 0.02 g DBTL, 0.06 g silicon oil as surfactant and 0.02 g water in a 40 mL glass vial (inner diameter 30 mm). Silicon oil was used, as a special surfactant could not be obtained commercially. The amount of MDI was calculated to 1:1.06 (OH:NCO) as to completely cure the foam, as shown in Table 7. The melted MDI was added, and the mixtures was mixed for 30 s. The reaction was then left until the foaming process was finished and left to cool down overnight. The glass vial was then carefully broken, and the finished foam was obtained.

### 2.4. Degradation of Monomers and Foams in Buffered Solution

Degradation studies have been performed on the monomers, as well as on the foams. The monomer degradation was monitored via ^31^P-NMR measurements. For this, 25 mg of the monomer was dissolved in 0.8 mL of an aqueous buffer solution at pH 7 (1 M, HEPES), 0.1 mL of THF and 0.1 mL of D_2_O. The samples were measured in regular intervals and stored at room temperature (RT). The degradation was determined via integrals. For this, the integral of the APtA peaks was divided by the integral of all peaks present in the spectra.

The foams were placed in approximately 50 mL of an aqueous buffer solution at pH 7 (PBS). After 7 days, the samples were taken out of the buffered solution, gently dried on a filter paper and the swelling was recorded. Then the foams were rinsed with water to remove any PBS residue and carefully dried in an oven at 40 °C, and the mass loss was recorded by weighing the dried foams. Then the samples were placed in approximately 50 mL of fresh buffered solution.

### 2.5. SEM Imaging

Scanning electron microscopy (SEM) was performed using a Xe-Plasma FIB (Tescan Amber X; Tescan GmbH, Libušina třída 863/2, 623 00 Brno, Czech Republic) operated at 5 keV and a beam current of 300 pA. The samples were sputtered with gold before imaging.

The cell size was measured using Digimizer.

### 2.6. Compression Testing

Dynamic mechanical analysis (DMA) was carried out using a TA DMA Q800 (TA Instruments, Helfmann-Park 10, 65760 Eschborn, Germany). DMA measurements of the set foams were performed in accordance with ISO 844 (ISO 844:2021, Rigid cellular plastics –determination of compression properties, ISO, 2021, Switzerland) at room temperature with foam disks (diameter: 30 mm, height: 8 mm). The method used a strain ramp of 5% min^−1^ (0.2 mm min^−1^) from 0 to 60% and back to 0% for a total of 2 cycles (strain rate).

## 3. Results and Discussion

**Figure 4:**
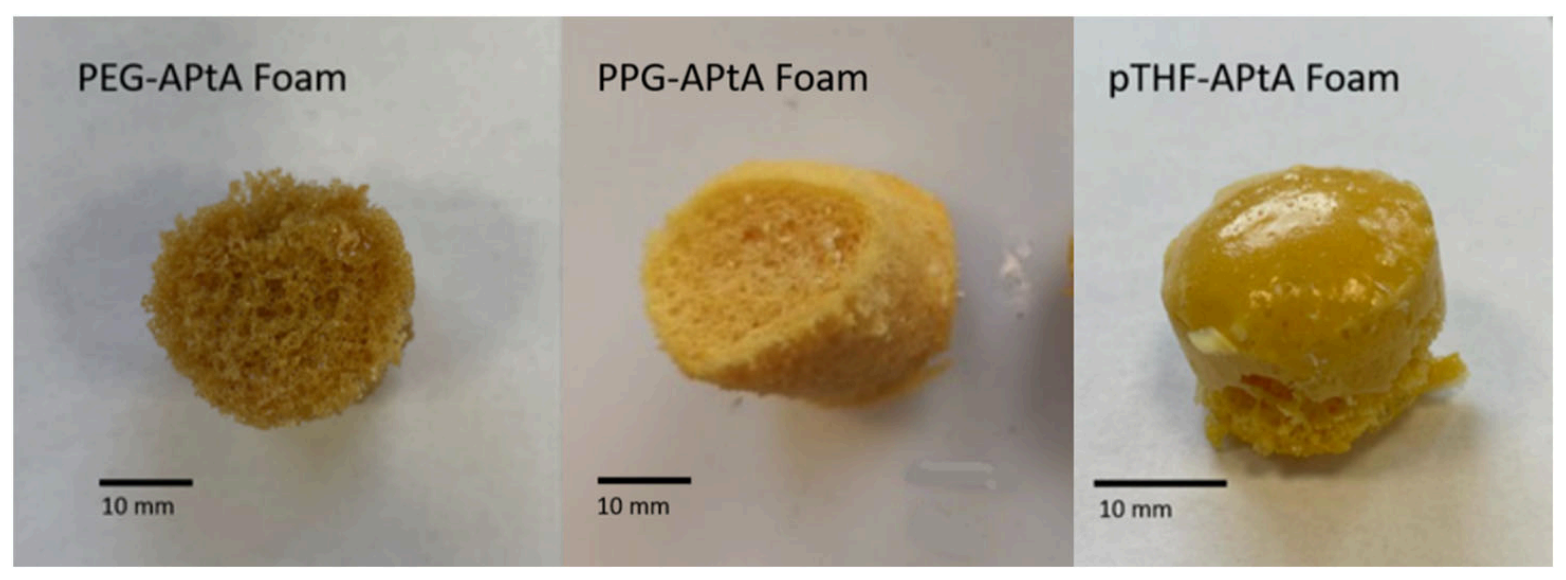
Overview of synthesized foams based on PEG-APtA, PPG-APtA and pTHF-APtA (left to right).

Three different PU foams were synthesized and characterized. Each foam was made out of a degradable monomer containing an APtA-based polyol (PEG, PPG or pTHF). The foams are pictured in Figure 4. The foams containing APtA-based polyols were all yellow, which came from the APtA-based polyols. Further, all foams looked like conventional PUR foams; they had open cell structures, were soft and flexible to touch, and their foam heights were similar.

Monomer hydrolysis was observed using ^31^P-NMR spectroscopy in a buffered solution at pH = 7. At the beginning, there was only one peak visible in the NMR spectra at approximately 17 ppm for all three monomers. After seven days, a second signal at 0 ppm could be observed, indicating a degradation from the APtA monomer into phosphates and glycine–polyol chains.

**Figure 5:**
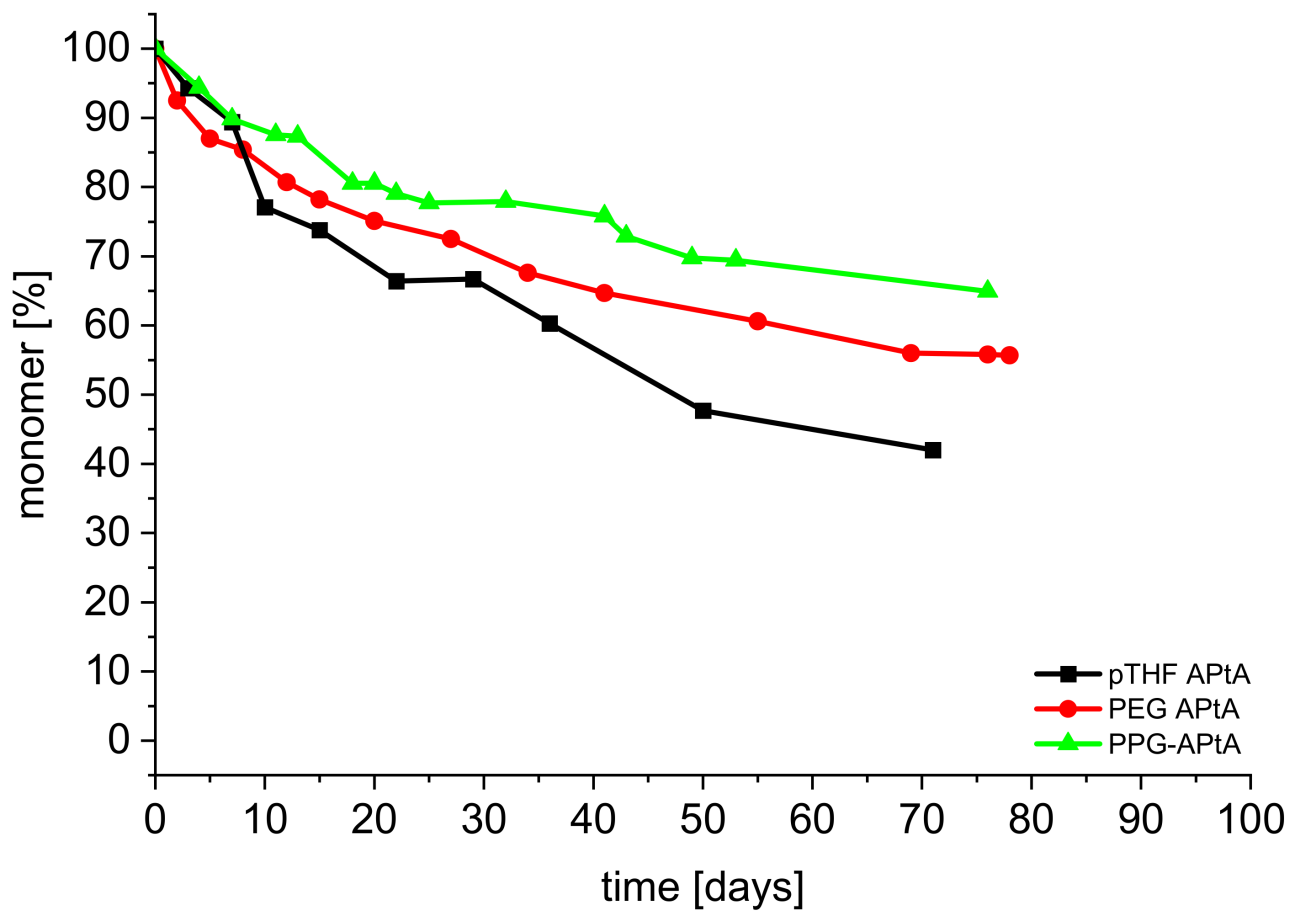
Hydrolysis of APtA-based monomers in buffered solutions at pH 7, measured via ^31^P-NMR. Peaks at 17 ppm were integrated and represent the APtA monomer.

The hydrolysis of the monomers followed a one-step degradation; no intermediate signals could be observed. This is in contrast to the degradation of difunctional amino acid phosphorodiamidates (APdA), where a stable intermediate could be observed using NMR spectroscopy [15,17]. The degradation of the synthesized monomers is plotted in Figure 5. At the beginning, all monomers degraded similarly fast, but after 30 days a trend emerged, showing that the pTHF-APtA monomer degraded slightly faster than the PEG-APtA and PPG-APtA monomers. After 30 days, 30% of the pTHF-APtA monomer was hydrolyzed, while only 25% of the PEG-APtA monomers and 20% of the PPG-APtA monomers were hydrolyzed. After 70 days, over 50% of the pTHF-APtA monomer was degraded, while PPG-APtA was at 35% monomer degradation and PEG-APtA was at 40%. There is no indication that the hydrophobicity or hydrophilicity of the polyol chain affected the degradation behavior of the monomers, as the hydrophilic PEG-APtA degraded slower than the more hydrophobic PPG-APtA, while the most hydrophobic, pTHF-APtA, degraded the fastest.

All synthesized APtA foams underwent a visual change during degradation. The PEG-APtA foam degraded rapidly and broke down into smaller fragments. The color of the foam stayed unchanged. For the pTHF-APtA, only small visual changes could be observed, such as slight deformation. After 70 days, a subtle darkening of the pTHF-APtA foam was observed. The PPG-APtA foam gradually decreased in size during degradation and exhibited a noticeable color change, transitioning from yellow to a lighter, off-white yellowish tone.

**Figure 6:**
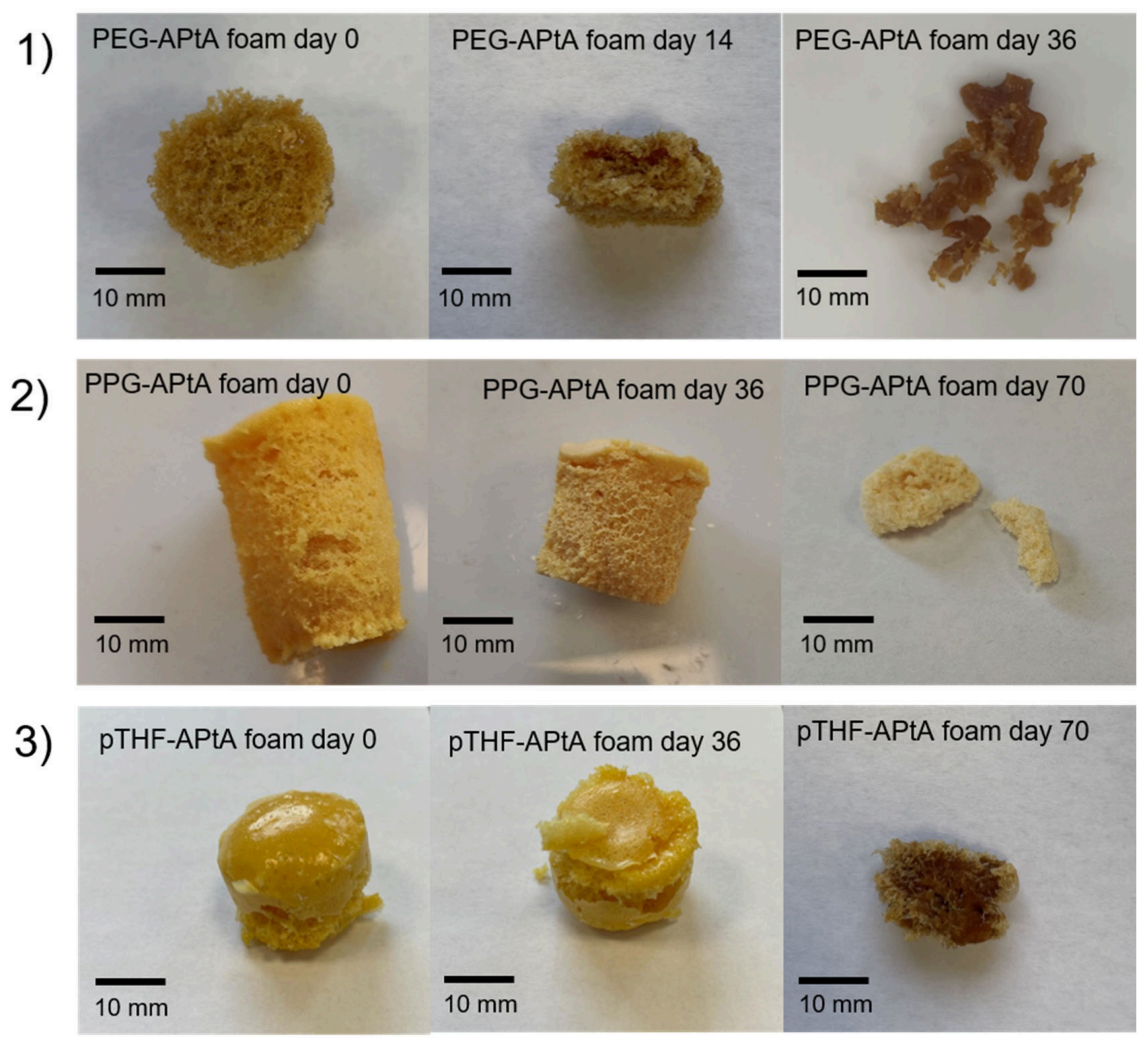
Pictures of all foams during degradation. (**1**) shows the PEG foam after 0, 14 and 36 days respectively: (**2**) shows the PPG-APtA foam after 0, 36 and 70 days; (**3**) shows the pTHF-APtA foam after 0, 36 and 70 days.

**Figure 7:**
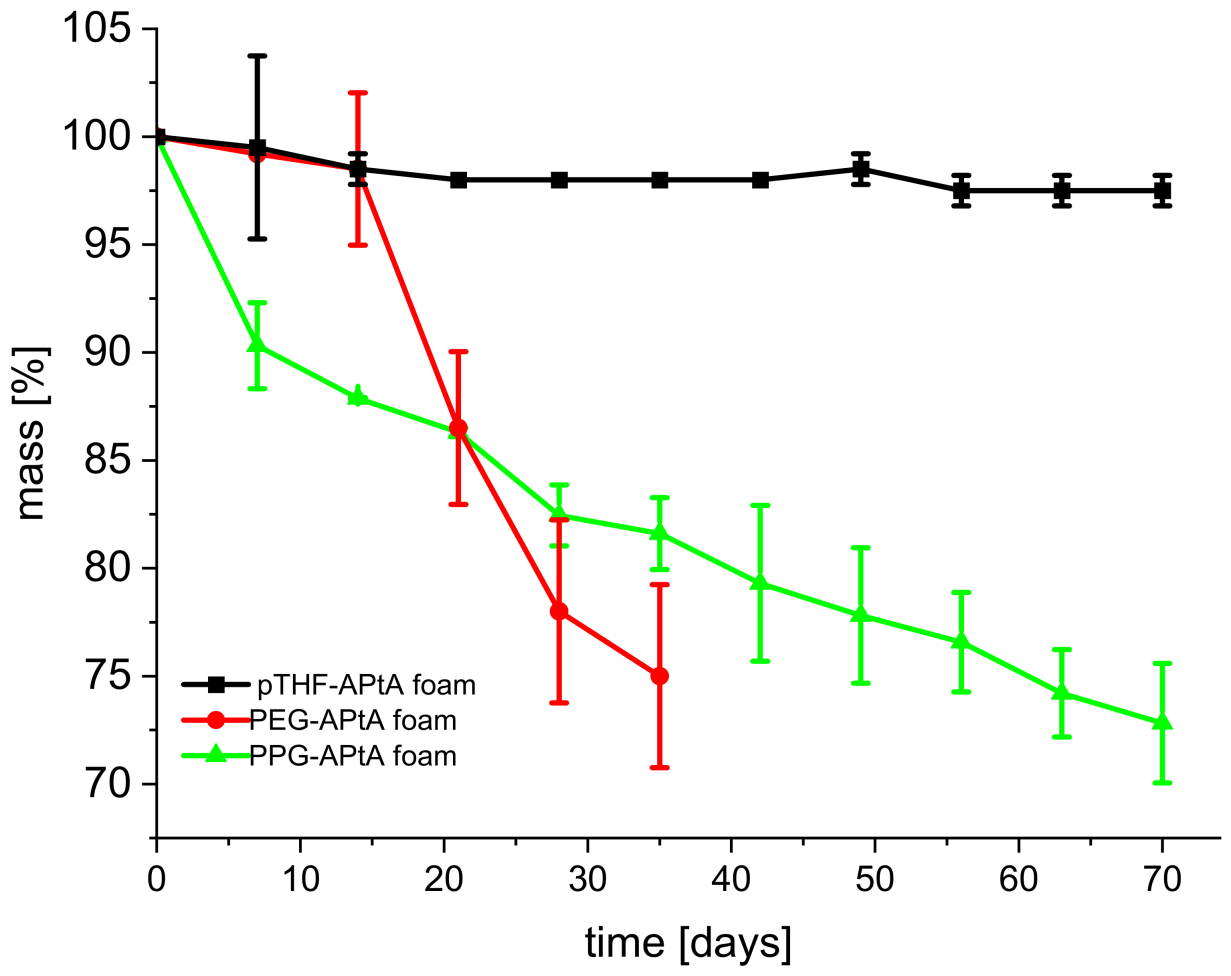
Degradation of PEG-APtA (red), PPG-APtA (green) and pTHF-APtA (black) foams in buffered solutions (PBS) at pH 7. Samples were measured in duplicates.

The hydrolytic degradation and mass loss of the foams correlates with the visual degradation as seen in Figure 6. Here, the foam based on the PEG-APtA degraded considerably faster than the foam based on pTHF-APtA or PPG-APtA. This can be seen by the change in the PEG-APtA foam appearance: the foam shrinks and deforms as the degradation progresses further. PEG is more hydrophilic than pTHF, so the water uptake into the foam is higher, and thus the APtA breakage point hydrolyzed faster. The foam containing pTHF-APtA or PPG-APtA shows a lower water uptake and the APtA functionality is thus hydrolyzed slower, as shown in Figure 7.

**Figure 8:**
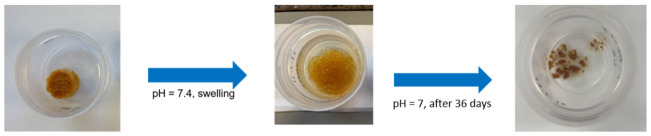
Observed swelling of the PEG-APtA foam in a buffered solution (PBS) at pH 7. Left: dry foam; center: foam after 7 days in the buffered solution; right: foam after 36 days (dried).

The PEG-APtA foam showed a slow degradation with a low mass loss for the first 14 days; after that, the PEG-APtA foam degraded faster than the other two foams, showing a mass loss of 25% in 30 days. This led to a complete disintegration of the foam network, which can be seen in Figure 8. The PPG foam degraded faster than the PEG foam for the first 21 days, showing a mass loss of 10% in the first week. After 21 days, the mass loss of both the PEG-APtA and PPG-APtA foams was 16%. After that, the PPG-APtA foam continuously degraded, albeit slower than the PEG-APtA foam, having lost about 20% mass after 30 days. After 70 days, the mass loss was at 26%. The foam shrunk considerably since the start of the degradation studies and started to disintegrate. The pTHF foam was nearly unchanged, having lost only 2% of its mass after 30 days and 2.5% after 70 days. This is in contrast to the monomer hydrolysis shown in Figure 5, where the pTHF-APtA monomer degraded quite fast, with over 50% of the monomer being hydrolyzed after 70 days. A reason for this difference in mass loss could be the hydrophobicity of the pTHF-APtA monomer. This is something that needs to be considered with the PPG-APtA monomer, as PPG is slightly hydrophobic. As the APtA bonds slowly break down, the hydrophobic pTHF and PPG chains seem to aggregate together, slowing down the hydrolysis of the foam network, while the PEG chains are water soluble, leaving the foam open to further hydrolysis, which results in a faster degradation; this can be observed in Figure 7.

**Figure 9:**
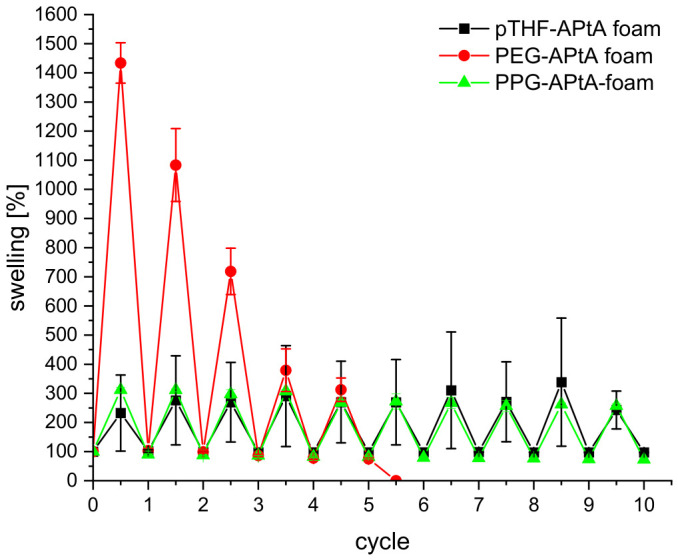
Swelling and mass loss of the foams. Each cycle comprised 7 days in a buffered solution (PBS) at pH 7. After each cycle, the foams were weighed and the water uptake was recorded; following this, the foam was dried and the mass loss was measured. Swelling tests were measured in duplicates.

The swelling behavior of the foams was also observed. After removing the foam samples from the buffer solution, the foams were weighted and the water uptake measured. The foams were taken out of the buffer and gently dried using filter paper; following this, the mass was determined. The foams were then dried. There is a trend between the mass loss of the foam and the water uptake. As seen in Figure 9, the foam containing PEG-APtA showed the highest water uptake with nearly 15 times of the original weight (red curve). The swelling of the PEG-APtA foam is shown in Figure 8 (center). The water uptake decreased with each cycle, correlating with the mass loss and degradation of the network of the foam. The PPG and pTHF-APtA foams (green and black curves, respectively) showed a lower water uptake, being only 3× and 2× of the foam weight, respectively. The water uptake of the PPG-APtA foam slowly decreased with each cycle (Figure 9) as the foam slowly degraded. After five cycles, the foam had lost 20% of its weight and still took up 85% of the water compared to the first cycle. After 10 cycles the PPG-APtA foam began to break into smaller pieces as the crosslinking points continued to degrade (pictures in Supplementary Information). The pTHF-APtA-based foam on the other hand showed the opposite trend. With a water uptake of only 2.5× in the first cycle, the water uptake only slightly increased during further cycles with a maximum uptake of 3.8× of the starting foam weight in cycle 8, while the mass of the foam stayed nearly unchanged. Additionally, the pTHF-APtA foam degraded slower than the PEG-APtA and PPG-APtA foam (Figure 7), which was probably caused by the hydrophobicity of the pTHF-chain. This strong hydrophobicity drastically slowed down the hydrolysis of the APtA-backbone and thus a slower degradation was observed. However, as the foam slowly degraded, the crosslinked network opened up and more water was taken up by the foam structure.

NMR spectroscopy of the degraded PEG-APtA foam leftovers showed no leftover phosphorous peaks of the PEG-APtA monomer or any degradation products after 36 days. The main degradation product, phosphoric acid and the salts thereof, are water-soluble and would be removed with the buffer after each cycle. The spectra can be found in the Supplementary Information.

**Figure 10:**
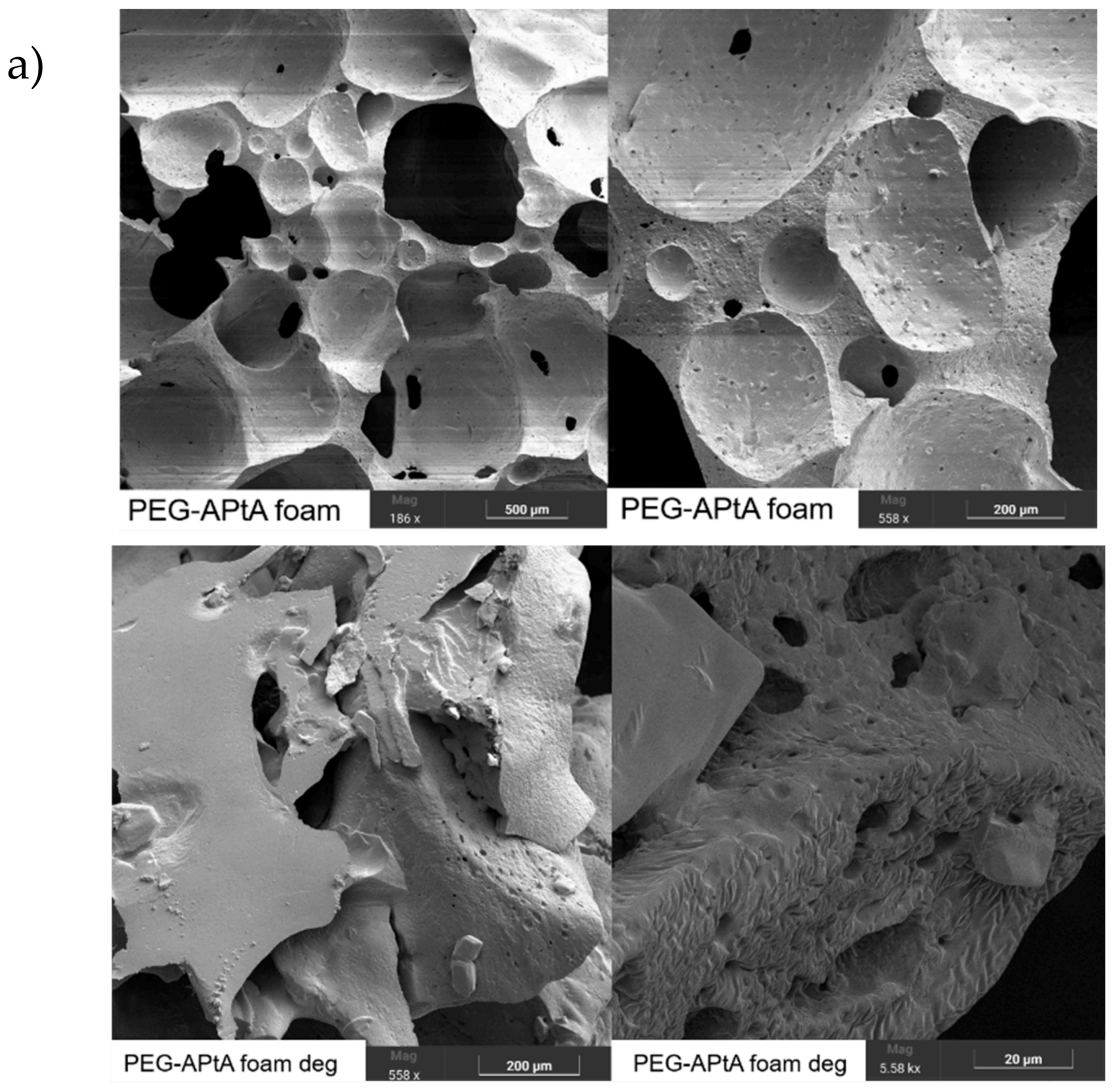

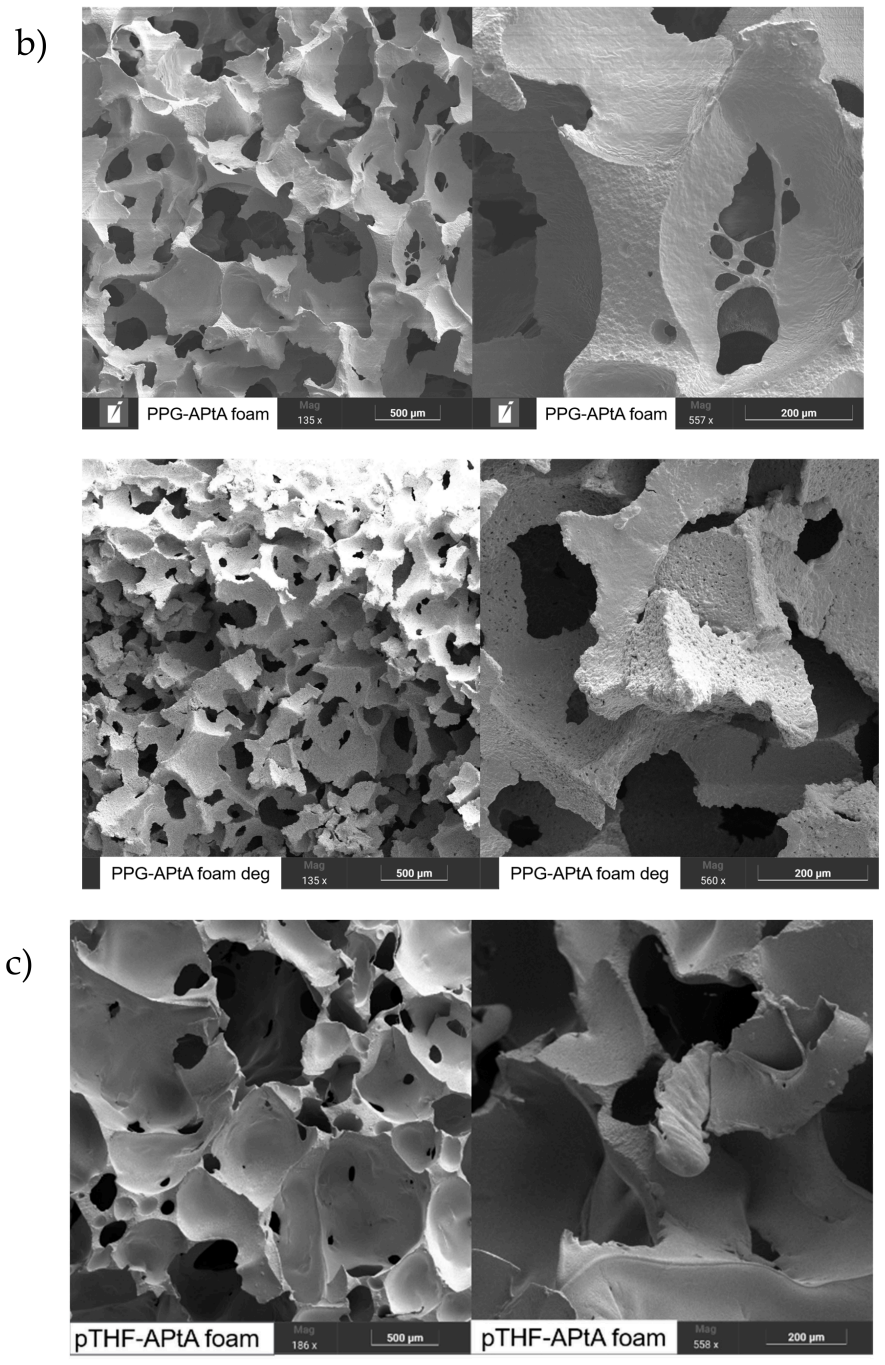

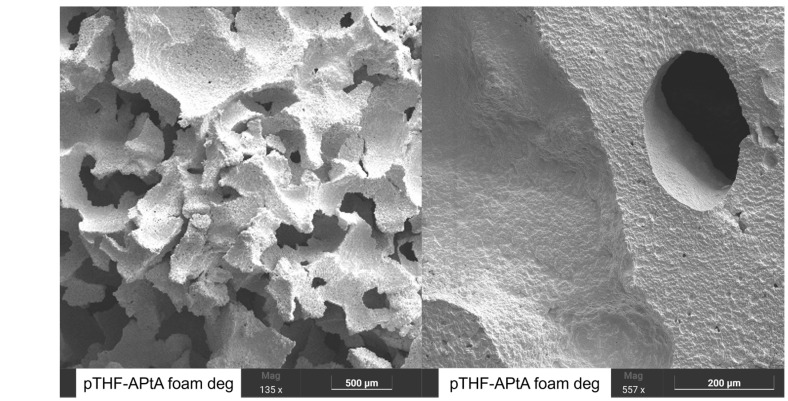
SEM images of PEG-APtA (**a**), PPG-APtA (**b**) and pTHF-APtA (**c**) fresh foam images are in the top row. Images of degraded samples are in the bottom. The images are at 186× and 568× (left and right, respectively) resolutions.

SEM images of the foams were taken. The pictures can be seen in Figure 10. The foam structures between the three different foams look quite similar and all appear with an open cell structure. The average cell size is presented in Table 8. The foams show an average cell size of about 550 µm with a very high standard deviation (SD) of ±50%. This is mostly due to the application of a PDMS silicon oil as a (not ideal) foam stabilizer. Dedicated foam stabilizers were not used due to a lack of availability. The application of a dedicated foam stabilizer could have yielded smaller cells with an even cell size distribution. Foam stabilizers are typically based on polydimethylsiloxane modified with EO/PO pendant groups, which act as surfactants during the foaming process. They reduce surface tension, stabilize the cell membranes, and promote a more uniform distribution of foam cells. The big and uneven cell structure in our experiments might have affected the mechanical properties of the APtA foams negatively. After degradation, the foam structures looked different. This was especially pronounced in the sample of the PEG-APtA foam, which disintegrated into small pieces (Figure 8). The open cell foam structure collapsed upon itself as the crosslinking points degraded. However, the PEG-APtA foam structural changes were less pronounced, as seen in the SEM images (Figure 10); most noticeable was that the edges became jagged and rougher during the degradation period and the whole networks appeared to be denser, which was also observed with the shrinking of the foam. The pTHF-APtA foam looked unchanged after degradation.

**Figure 11:**
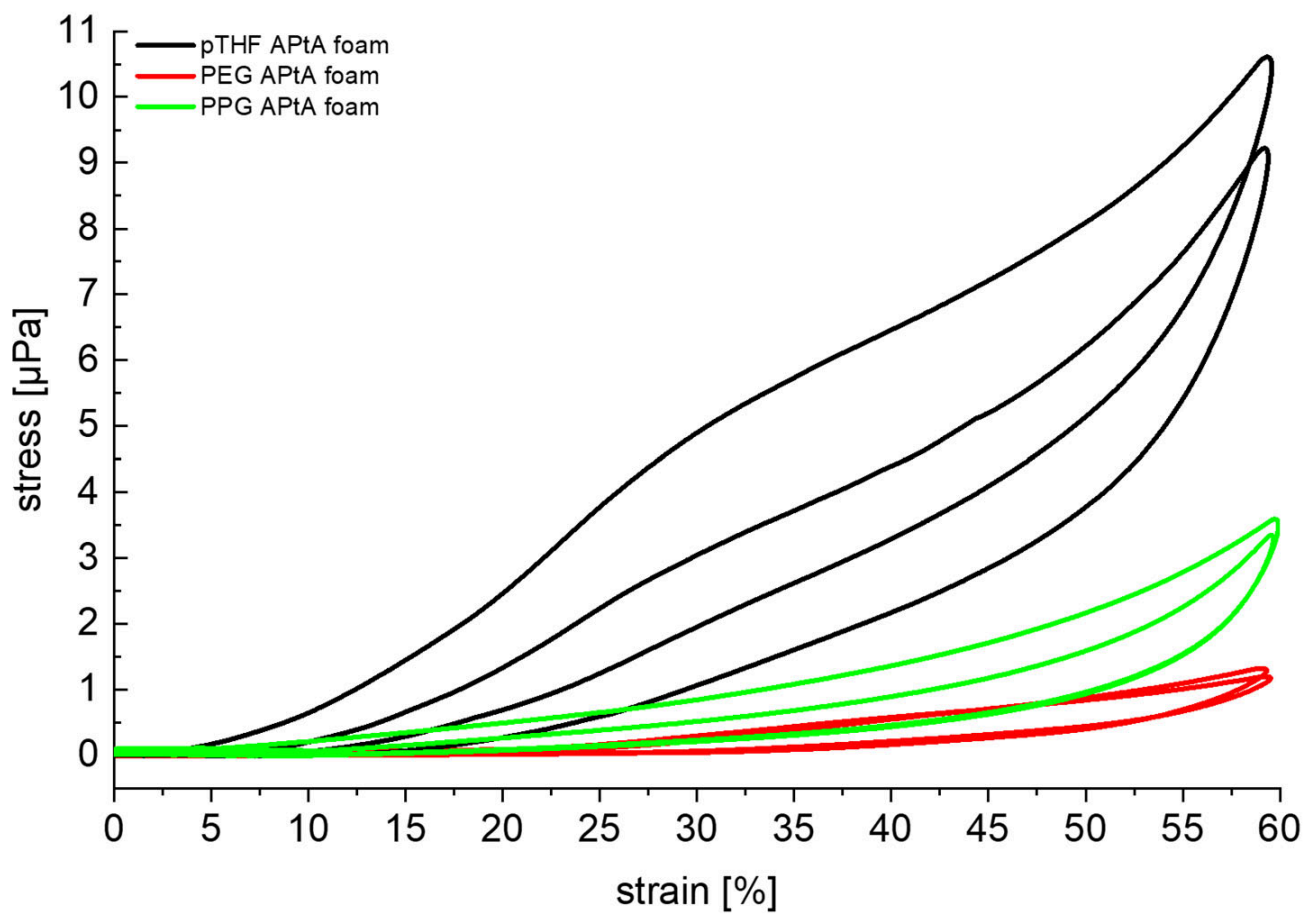
DMA measurements of the degradable APtA-based foams with fixed strain rate (0.2 mm/min), up to 60% strain.

Mechanical analyses of the APtA foams were done using compression measurements in accordance with ISO 844. The obtained stress–strain curves are shown in Figure 11.

Both the PEG-APtA and PPG-APtA foams exhibited typical soft PUR foam behavior, with a nearly linear elastic plateau up to 50% strain, followed by an increase in stress up to the maximum strain of 60%. The pTHF-APtA foam showed a continuous increase in stress with increasing stain without a linear elastic plateau. This was because of the stiffer backbone of pTHF, which required more stress to compress, as there were four carbon atoms between the ether linkages compared to two with PEG and PPG.

**Figure 12:**
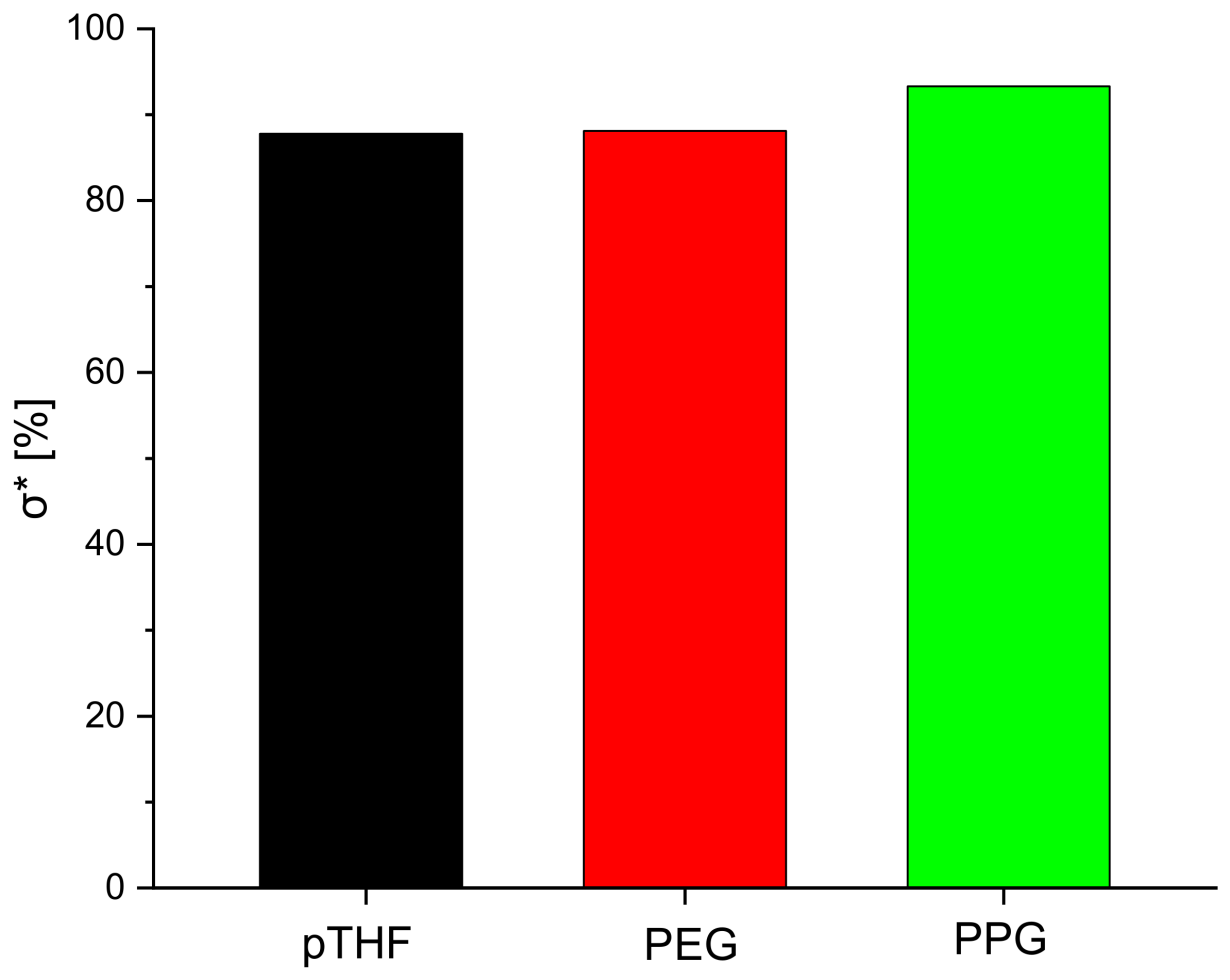
Stress softening (σ*) of TPU foam under 60 % strain.

**Figure 13:**
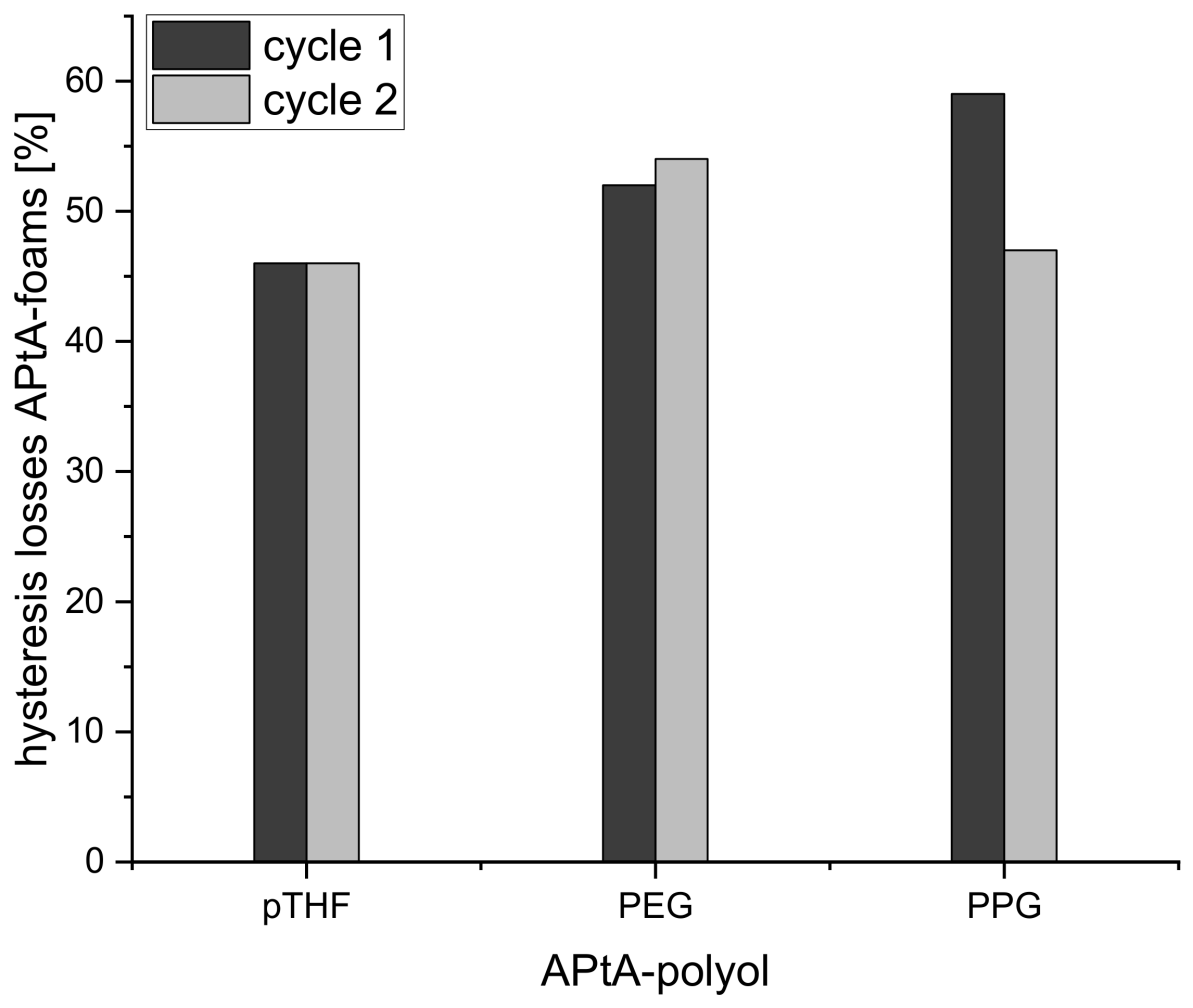
Hysteresis losses of APtA foams during compression measurements in %.

From the DMA data the stress softening (σ*) and the hysteresis losses were calculated and are shown in Figure 12 and Figure 13, respectively.

As seen in Figure 12 and Figure 13, the values of σ* were relatively low and the hysteresis loss was very high in comparison to commercially available soft PUR foams. Typically, σ* is at values of above 98% at one cycle, while hysteresis losses are mostly below 20% [21,22]. This means that the APtA foams showed a low elasticity and high permanent deformation in comparison to commercial soft PUR foams. This suggests that the foam structure was not highly crosslinked or uneven, as could be seen in the SEM images, which allowed the permanent deformation to occur. As our foams were synthesized from only one APtA monomer each and foamed with water, without the application of dedicated foam stabilizers, this behavior was expected. Commercial PUR foams use a blend of polyols with different chemical structures, chain lengths and functionalities, as well as additives such as stabilizers, surfactants and crosslinkers, to achieve better elasticity and higher resilience [23,24,25,26].

## 4. Conclusions

This work shows the preparation and characteristics of soft degradable polyurethane foams based on amino acid phosphoramides (APtA). Three foams using monomers with different polyol structures have been prepared. PEG, PPG and pTHF were chosen as polyol backbones for the degradable APtA monomers.

All three foams showed an open cell structure and the PEG and PPG foams exhibited a broad viscoelastic plateau, which is commonly found for soft polyurethane foams. The mechanical properties of the foams were similar, but all foams showed low stress relaxation and high hysteresis losses. This is partly explained by the uneven open cell structure of the foams. Further mechanical testing would need to be done to further characterize these foams for possible applications such as degradable pillows or mattresses.

The pTHF-APtA monomer showed a slightly faster hydrolysis in water than the PEG-APtA and PPG-APtA monomers, respectively. But the hydrolysis behavior follows no apparent trend in terms of hydrophobic or hydrophilic polyol chains.

In contrast, the mass loss of the synthesized APtA-PUR foams shows a clear trend; at the beginning, the foam based on PPG-APtA had the highest mass loss, followed by the PEG-APtA foam and the pTHF-APtA foam. After three weeks, the PEG foam had nearly the same mass loss as the PPG foam. From this point on, the PEG foam degraded much faster than the PPG foam, which may be explained by the higher water uptake, which in turn was possibly due to the hydrophilic PEG chains. A higher water uptake can result in a faster hydrolysis of the APtA monomers. The faster degradation of the PPG-APtA foam at the beginning may be explained by a slightly bigger pore size, which allowed for faster hydrolysis. This also explains the slow degradation of the hydrophobic pTHF foam, which only showed a low mass loss of 2.5% during the observation period and a lower water uptake than the PEG foam. The PPG foam showed a constant mass loss during the observation period.

Overall, this novel work demonstrates how APtA-based monomers can be incorporated into soft polyurethane foams and that these foams can degrade under mild conditions in a timely manner.

## Figures and Tables

**Table 7:** Recipes of foams.

Name	Monomer	[g]	MDI [g]
Reference	Glycerol propopxylate (M_w_ = 3600)	2.0	0.5153
GlyPEG	Gly-PEG-ApTA	2.0	0.5376
GlyPPG	Gly-PPG-APtA	2.0	0.5376
GlypTHF	Gly-pTHF-APtA	2.0	0.5376

**Table 8:** Average cell size of synthesized APtA foams [n = 20] and standard deviation (SD).

	Cell Size [µm]	SD [µm]
PEG-APtA foam	550	±227
PPG-APtA foam	625	±146
pTHF-APtA foam	555	±221

## Data Availability

The original contributions presented in this study are included in the article/Supplementary Material. Further inquiries can be directed to the corresponding author.

## Supplementary Materials

The following supporting information can be downloaded at https://www.mdpi.com/article/10.3390/polym18121534/s1.

## Abbreviations

The following abbreviations are used in this manuscript:

APtA	Amino acid phosphoramide
PUR	Polyurethane
TPU	Thermoplastic polyurethane
EO	Ethylene oxide
PO	Propylene oxide
APdA	Amino acid phosphordiamidates
PEG	Polyethylene glycol
PPG	Polypropylene glycol
pTHF	Polytetrahydrofuran
NMR	Nuclear magnetic resonance
SEM	Scanning electron microscope
PDMS	Poly dimethylsiloxane
DMA	Dynamic mechanical analysis
SD	Standard deviation

## References

[1] Ates M., Karadag S., Eker A.A., Eker B. (2022). Polyurethane foam materials and their industrial applications. Polym. Int..

[2] Banik J., Chakraborty D., Rizwan M., Shaik A.H., Chandan M.R. (2023). Review on disposal, recycling and management of waste polyurethane foams: A way ahead. Waste Manag. Res..

[3] Madbouly S.A. (2023). Novel recycling processes for thermoset polyurethane foams. Curr. Opin. Green Sustain. Chem..

[4] Deng Y., Dewil R., Appels L., Ansart R., Baeyens J., Kang Q. (2021). Reviewing the thermo-chemical recycling of waste polyurethane foam. J. Environ. Manag..

[5] Grdadolnik M., Zdovc B., Drinčić A., Onder O.C., Utroša P., Ramos S.G., Ramos E.D., Pahovnik D., Žagar E. (2023). Chemical Recycling of Flexible Polyurethane Foams by Aminolysis to Recover High-Quality Polyols. ACS Sustain. Chem. Eng..

[6] Grdadolnik M., Drinčić A., Oreški A., Onder O.C., Utroša P., Pahovnik D., Žagar E. (2022). Insight into Chemical Recycling of Flexible Polyurethane Foams by Acidolysis. ACS Sustain. Chem. Eng..

[7] Savelyev Y., Markovskaya L., Savelyeva O., Akhranovich E., Parkhomenko N., Travinskaya T. (2015). Degradable polyurethane foams based on disaccharides. J. Appl. Polym. Sci..

[8] Serrano L., Rincón E., García A., Rodríguez J., Briones R. (2020). Bio-Degradable Polyurethane Foams Produced by Liquefied Polyol from Wheat Straw Biomass. Polymers.

[9] Huang S.J., Roby M.S. (1986). Biodegradable Polymers Poly(amide-urethanes) [1]. J. Bioact. Compat. Polym..

[10] Furtwengler P., Avérous L. (2018). Renewable polyols for advanced polyurethane foams from diverse biomass resources. Polym. Chem..

[11] Skleničková K., Abbrent S., Halecký M., Kočí V., Beneš H. (2022). Biodegradability and ecotoxicity of polyurethane foams: A review. Crit. Rev. Environ. Sci. Technol..

[12] Tokiwa Y., Calabia B.P., Ugwu C.U., Aiba S. (2009). Biodegradability of plastics. Int. J. Mol. Sci..

[13] Shi L., Gong Z., Xie M., Shao W., Cheng H., Gao S. (2026). Photodegradation behavior of different polyurethane: A comparison study of foam and leather. J. Environ. Sci..

[14] Branson Y., Söltl S., Buchmann C., Wei R., Schaffert L., Badenhorst C.P.S., Reisky L., Jäger G., Bornscheuer U.T. (2023). Urethanasen für die enzymatische Hydrolyse niedermolekularer Carbamate und das Recycling von Polyurethanen. Angew. Chem..

[15] Vennemann N., Hubner F., Haudum S., Teasdale I., Brüggemann O. (2026). Degradable polyurethanes with amino-acid phosphorodiamidates as breakage points. Monatshefte Für Chem.-Chem. Mon..

[16] Mostofizadeh M., Kainz M., Alihosseini F., Haudum S., Youssefi M., Bauer P., Gnatiuk I., Brüggemann O., Zembsch K., Rinner U. (2024). Phosphoramide Hydrogels as Biodegradable Matrices for Inkjet Printing and Their Nano-Hydroxyapatite Composites. ACS Appl. Mater. Interfaces.

[17] Haudum S., Demirdögen B., Müller-Müchler L., Döttl S.C., Müller S.M., Naderer C., Brüggemann O., Griesser T., Jacak J., Priglinger E. (2024). Biodegradable resins for photochemical 3D printing via vinyl ester and vinyl carbonate functionalized amino acid-phosphoramidates. Eur. Polym. J..

[18] Herzberger J., Niederer K., Pohlit H., Seiwert J., Worm M., Wurm F.R., Frey H. (2016). Polymerization of Ethylene Oxide, Propylene Oxide, and Other Alkylene Oxides: Synthesis, Novel Polymer Architectures, and Bioconjugation. Chem. Rev..

[19] Maamoun A.A., Arafa M., Esawi A.M.K. (2025). Flexible polyurethane foam: Materials, synthesis, recycling, and applications in energy harvesting—A review. Mater. Adv..

[20] Pinto M.L. (2010). Formulation, Preparation, and Characterization of Polyurethane Foams. J. Chem. Educ..

[21] Gao X., Niu C., Wang J., Wu Y., Chen Y., Zhao L., Hu D. (2025). Micro-crosslinked thermoplastic polyurethane foam with ultra-low density, excellent resilience and recycling by supercritical CO_2_/N_2_. J. CO_2_ Util..

[22] Abdullah M., Ramtani S., Yagoubi N. (2023). Mechanical properties of polyurethane foam for potential application in the prevention and treatment of pressure ulcers. Results Eng..

[23] Gama N.V., Ferreira A., Barros-Timmons A. (2018). Polyurethane Foams: Past, Present, and Future. Materials.

[24] Gu R., Konar S., Sain M. (2012). Preparation and Characterization of Sustainable Polyurethane Foams from Soybean Oils. J. Americ. Oil Chem. Soc..

[25] Zhang X.D., Macosko C.W., Davis H.T., Nikolov A.D., Wasan D.T. (1999). Role of Silicone Surfactant in Flexible Polyurethane Foam. J. Colloid Interface Sci..

[26] Zhang C., Tong X., Deng C., Wen H., Huang D., Guo Q., Liu X. (2020). The foaming dynamic characteristics of polyurethane foam. J. Cell. Plast..

