# Supplementary material for: Degradable Polyurethane Foams Based on Amino Acid Phosphoramides (APtA)
Source: Polymers (Basel). 2026 Jun 20;18(12):1534. doi: 10.3390/polym18121534 (PMC13307100; doi:10.3390/polym18121534)
Supplement: Supplementary file 1 [file polymers-18-01534-s001.zip › polymers-4363808-supplementary.pdf]

Supporting Information

Degradable polyurethane foams based on amino acid phosphoroamides (APtA)

Nico Vennemann[1] ● Anton Bauer[1] ● Oliver Brüggemann[1,*]

Table of Contents

Supporting Information 1

Degradable polyurethane foams based on amino acid phosphoroamides (APtA) 1

^{1}H- and ^{31}P-NMR spectra 3

^{1}H- and ^{31}P-NMR spectra

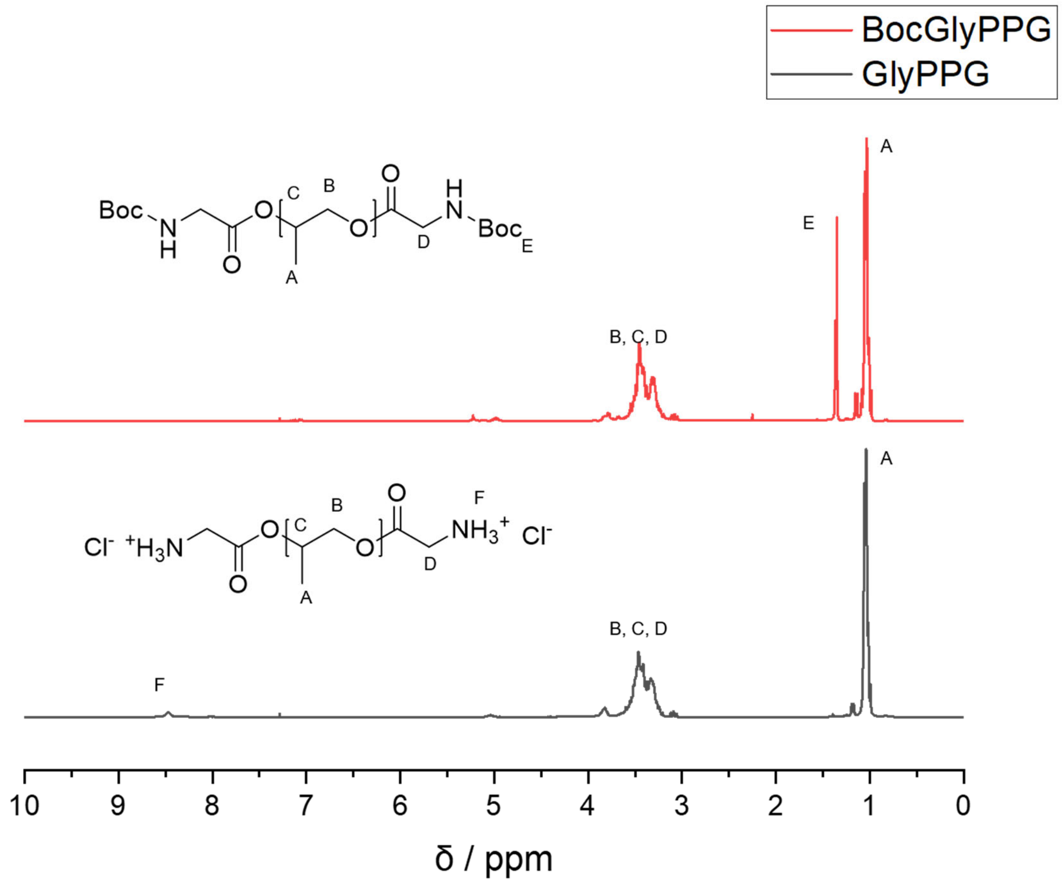

Figure S1. ^{1}H-NMR spectra of BocGlyPPG and GlyPPG.

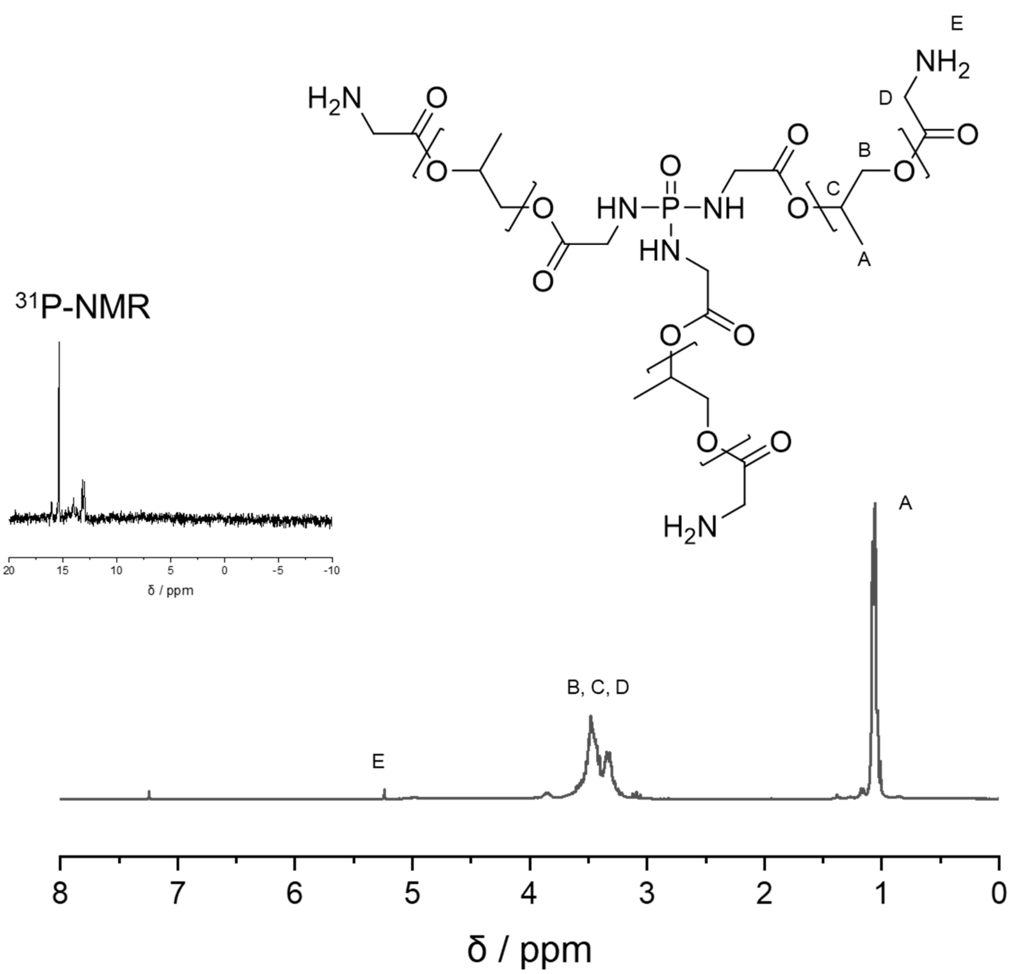

Figure S2. ^{1}H and ^{31}P-Spectra of Gly-PPG-APtA.

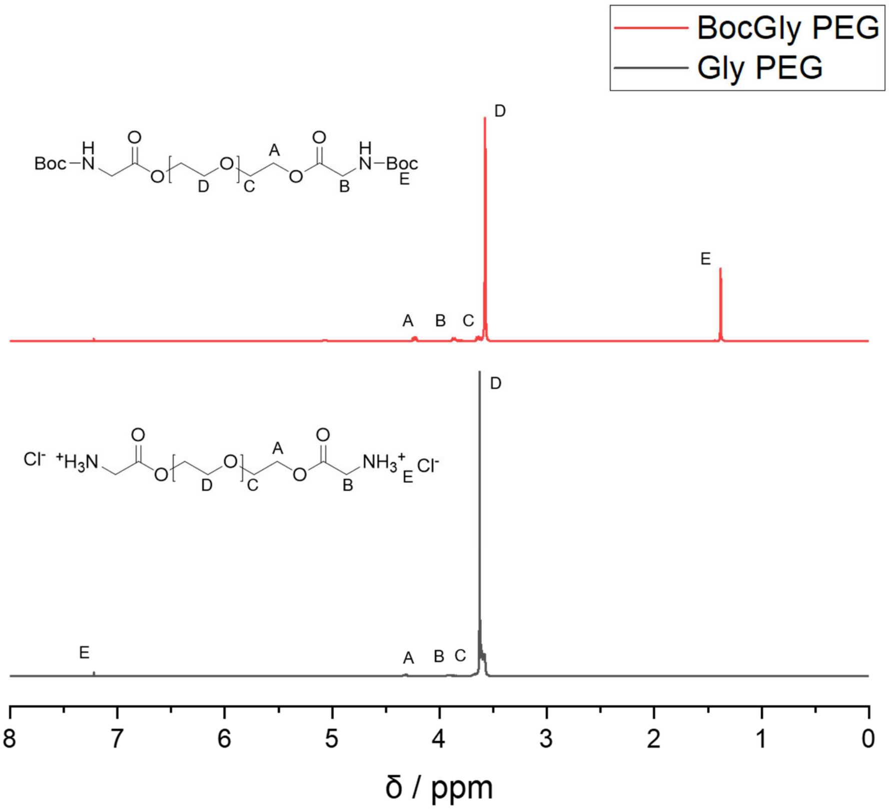

Figure S3. ^{1}H-NMR spectra of BocGlyPEG and GlyPEG.

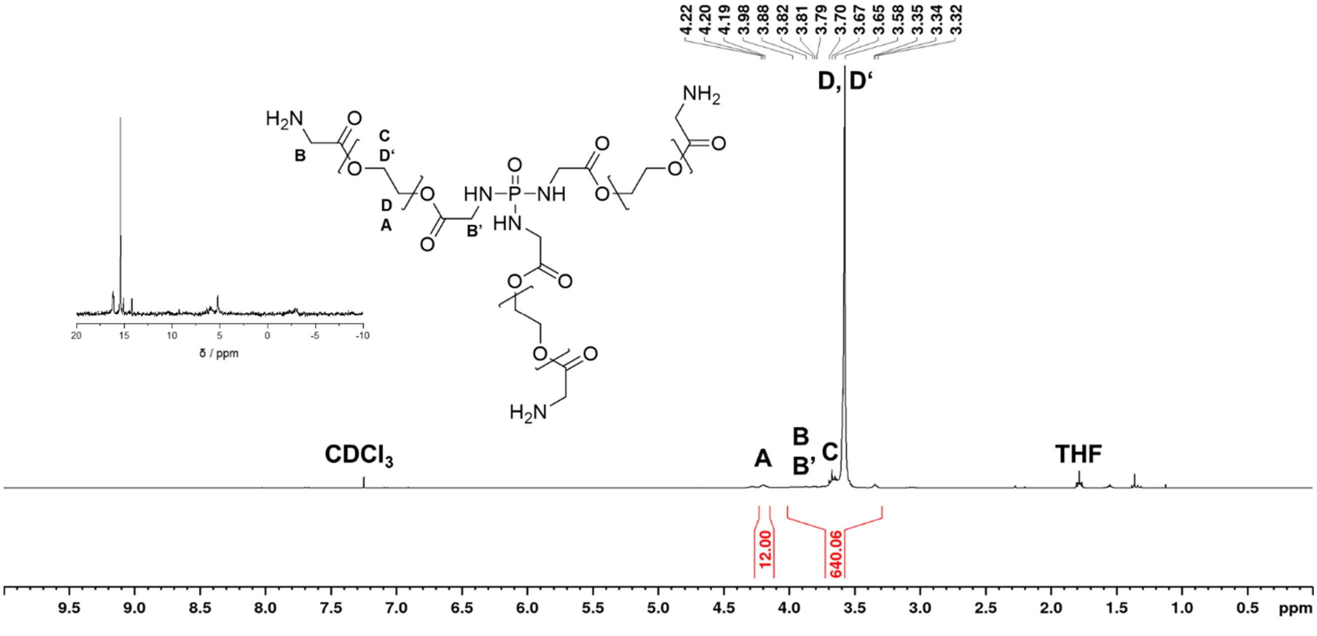

Figure S4. ^{1}H and ^{31}P-Spectra of Gly-PEG-APtA.

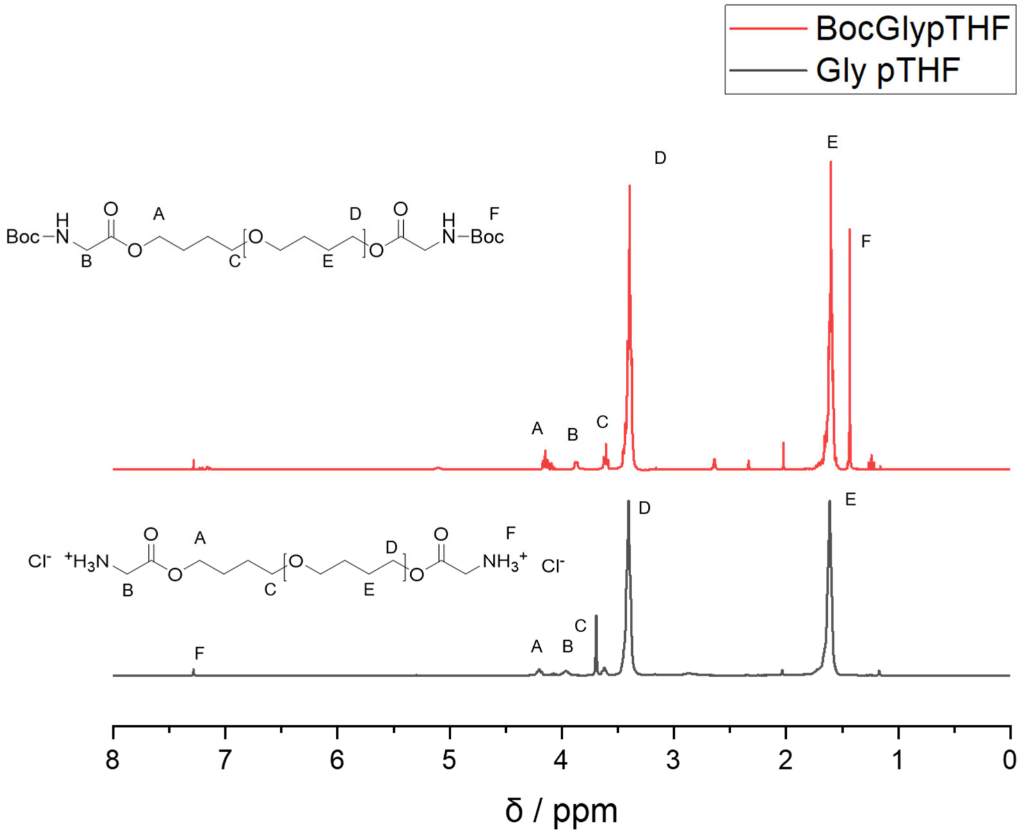

Figure S5. ^{1}H-NMR spectra of BocGlypTHF and GlypTHF.

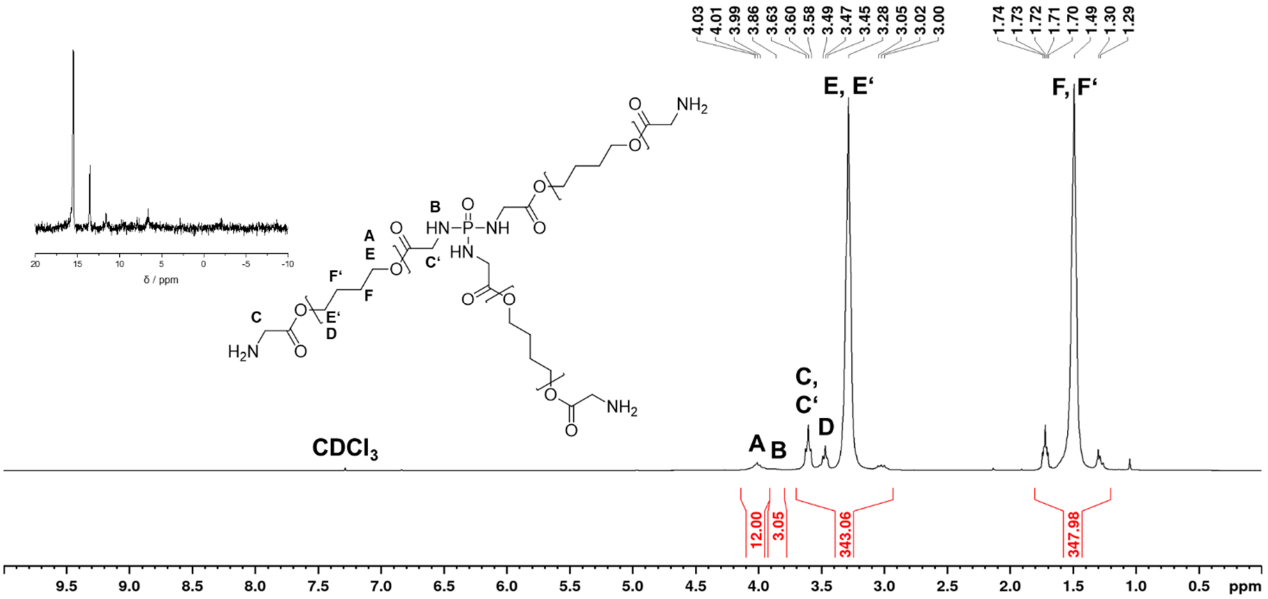

Figure S6. ^{1}H and ^{31}P-Spectra of Gly-pTHF-APtA.

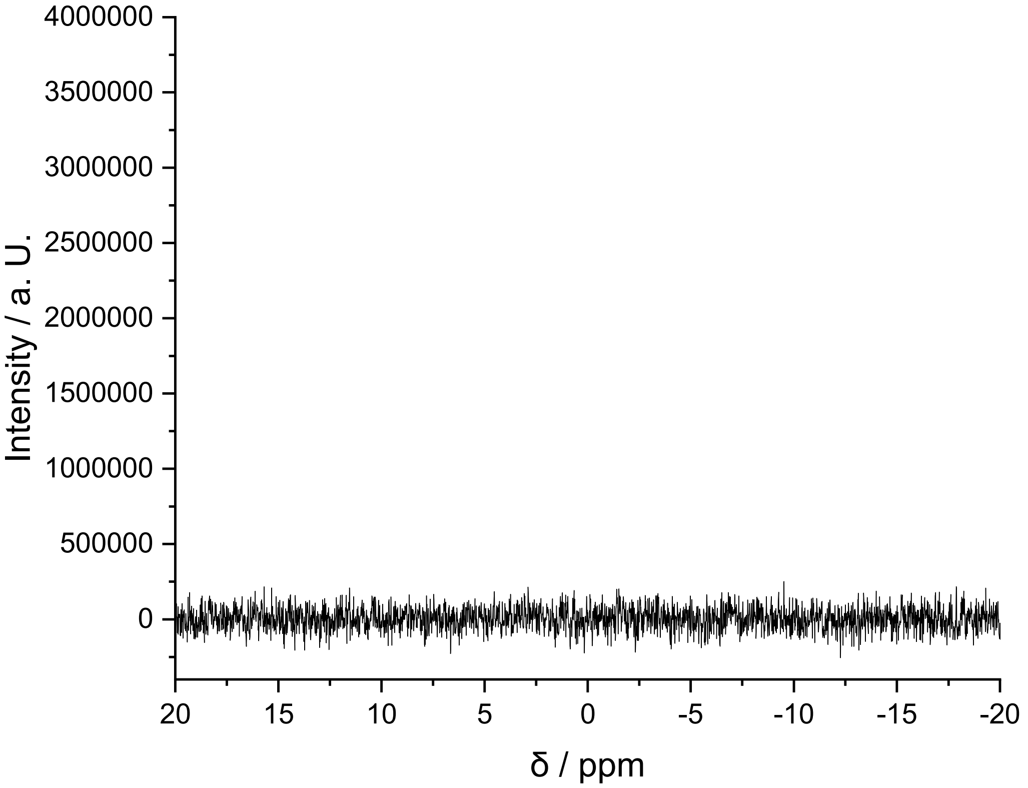

Figure S7. ^{31}P-NMR spectrum of degraded APtA-PEG foam, there is no phosphorous signal visible, indicating degradation of the APtA bonds.
